# Case of Poisoning by Vinegar

**Published:** 1847-11

**Authors:** A. H. David

**Affiliations:** Montreal


*Case of Poisoning by Vinegar.* By A. H. David, M. D., Montreal. —Poisoning by acetic acid is so uncommon an occurrence, I have to request a small portion of your valuable columns for the details of a case I met with a few days ago; in which the patient—a widow woman, with four children—took, as near as I could ascertain, a quart bowlful of common vinegar. It appears that she had been dull and low spirited for two or three days previous, in consequence of the neglect (as her friends suppose) of a person from whom she had received the most marked attention, and to whom she had been attached prior to her marriage with her late husband. When I saw her, about three hours after she had taken the vinegar, she was in bed, covered with a cold perspiration, and trembling from head to foot, and apparently alarmed at everybody and everything about her. Her breathing was very laborious and hurried ; her countenance perfectly wild, and the pupils dilated; the tongue was dry and cold ; pulse ninety-six and full : the abdomen much distended, with extreme acute pain at the *scrobiculis cordis*, so much so, that the slightest pressure there caused her to shriek out. She did not know any one about her, not even her own children, nor had she any recollection of anything that had happened from the time of taking the vinegar, which was about 11 at night, not even of her having gone to bed, which she was the last in the house to do. About 1 o'clock the inmates were all awakened by her shrieking for cold water, of which she had drunk an enormous quantity before I was called to see her. There was not any pain, heat, or constriction of the throat or fauces, but there were slight efforts to vomit. Having procured some sulphate of zinc, I gave her two scruples in a cup of water, which soon produced full vomiting, with great straining. I had then to leave her, but ordered full and repeated doses of carb. magnesia, till I could see her again, which I did about six hours after, and found her much relieved, and only complaining of headache, which left her after the operation of a dose of castor oil. Two days after, she was taken ill with a slight attack of continued fever, but is doing well.

I should mention that the quantity she threw up from the effects of the zinc was very great, and smelt strongly of vinegar, which she still perseveres in saying she did not take, although she was seen with the bowl filled with it in her hands by some of the family, when they were retireing to rest, she maintaining that she used the whole of the vinegar in bathing her head. However, I think we have strong presumptive evidence against her having so used it, and are justified in concluding that she took the whole of it.

The only case of poisoning by acetic acid that I have been able to find, is the one related by Orfila in the Annales D'Hygiene, and quoted by both Beck and Christison. The experiments instituted by Orfila prove that common vinegar, in large quantities, was found destructive to dogs when vomiting was prevented.

Taylor, in his work on Medical Jurisprudence says, " Acetic, citric and tartaric acids are not commonly considered to have any poisonous action on the body. At least, as far as I know, there is no case reported of their having acted injuriously on the human subject ; and he is the only modern writer on medical jurisprudence who takes any notice or makes mention of acetic acid.—*British American Journal of Medical and Physical Science.*

